# The functional microscopic neuroanatomy of the human subthalamic nucleus

**DOI:** 10.1007/s00429-019-01960-3

**Published:** 2019-09-28

**Authors:** Anneke Alkemade, Gilles de Hollander, Steven Miletic, Max C. Keuken, Rawien Balesar, Onno de Boer, Dick F. Swaab, Birte U. Forstmann

**Affiliations:** 1 Integrative Model-Based Cognitive Neuroscience (IMCN) Research Unit, University of Amsterdam, Nieuwe Achtergracht 129B, Room G3.06, PO Box 15926, 1001 NK Amsterdam, The Netherlands; 2 Laboratory for Social and Neural Systems Research (SNS-Lab), Department of Economics, University of Zurich, Zurich, Switzerland; 3 Spinoza Centre for Neuroimaging, Royal Academy of Sciences, Amsterdam, The Netherlands; 4 Department of Pathology, Location AMC of the Amsterdam UMC, Amsterdam, The Netherlands; 5 Department of Neuropsychiatric Disorders, An Institute of the Royal Netherlands Academy of Arts and Sciences, The Netherlands Institute for Neuroscience, Amsterdam, The Netherlands

**Keywords:** Subthalamic nucleus, Immunocytochemistry, Neuroanatomy, Basal ganglia

## Abstract

The subthalamic nucleus (STN) is successfully used as a surgical target for deep brain stimulation in the treatment of movement disorders. Interestingly, the internal structure of the STN is still incompletely understood. The objective of the present study was to investigate three-dimensional (3D) immunoreactivity patterns for 12 individual protein markers for GABA-ergic, serotonergic, dopaminergic as well as glutamatergic signaling. We analyzed the immunoreactivity using optical densities and created a 3D reconstruction of seven postmortem human STNs. Quantitative modeling of the reconstructed 3D immunoreactivity patterns revealed that the applied protein markers show a gradient distribution in the STN. These gradients were predominantly organized along the ventromedial to dorsolateral axis of the STN. The results are of particular interest in view of the theoretical underpinning for surgical targeting, which is based on a tripartite distribution of cognitive, limbic and motor function in the STN.

**Electronic supplementary material:**

The online version of this article (10.1007/s00429-019-01960-3) contains supplementary material, which is available to authorized users.

## Introduction

Among the over 450 subcortical structures of the human brain is the subthalamic nucleus (STN), which is involved in many functions, ranging from speeded decision-making to emotional regulation (Frank 2006; Herculano-Houzel 2012; Alkemade 2013; Péron et al. 2013; Aron et al. 2016; Forstmann et al. 2017), the STN is of particular interest as a target for deep brain stimulation (DBS) to alleviate symptoms in a variety of movement disorders including Parkinson’s disease (PD) (Temel et al. 2005).

The internal structure of the human STN is a topic of ongoing discussion and consistency between empirical studies is limited (Keuken et al. 2012; Alkemade et al. 2015). A prominent model of the internal structure of the STN in the scientific literature is the tripartite model, which divides the STN in a limbic medial tip, a ventromedial cognitive area, and a dorsolateral motor area (Temel et al. 2005). The level of anatomical separation between the subdivisions of the STN is unknown, and findings vary based on the applied research technique (Alkemade and Forstmann 2014; Lambert et al. 2015). The principle of functional segregation offers a theoretical framework for defining subdivisions in the STN at the cellular level. According to this principle, neuronal cell types move apart during development, depending on the specializations they acquire (Arendt 2008). During this process, they form distinct neuronal populations, with potentially distinct functions, as reflected by their individual molecular fingerprint. Immunocytochemical approaches in postmortem tissues allow the identification of neuronal subpopulations, and thereby potentially subdivisions within the STN (Forstmann et al. 2017).

The availability of detailed information on the immunocytochemical characteristics of the human STN is generally of high quality, but only a small number of detailed studies on serotonin (5HT), and its transporter (SERT), and on PARV and CALR expression report on a topographical organization within the STN (Mori et al. 1985; Parent et al. 1996, 2011; Augood et al. 1999). Additionally, studies on the human and nonhuman primate STN have revealed the expression of glutamatergic, GABA-ergic, dopaminergic, serotonergic as well as endogenous opioid markers, in addition to calcium-binding proteins (Kultas-Ilinsky et al. 1998; Augood et al. 1999, 2000; Hedreen 1999; Charara et al. 2000; Kuwajima et al. 2004; Levesque and Parent 2005; Aron and Poldrack 2006; Isoda and Hikosaka 2008). A number of the immunocytochemical studies available do not report specifically on distribution patterns within the human STN (Nauta and Cole 1978; Mori et al. 1985; Kultas-Ilinsky et al. 1998; Augood et al. 1999, 2000; Hedreen 1999; Charara et al. 2000; Hurd et al. 2001; Kuwajima et al. 2004; Levesque and Parent 2005; Aubert et al. 2007; Parent et al. 2011), and classical immunocytochemical studies generally are descriptive in nature. Here we set out to investigate the three-dimensional (3D) functional microscopic neuroanatomy of the human STN in a systematic manner, and allowing quantitative stereological analyses of the data. We have created 3D reconstructions of immunocytochemical staining patterns for quantitative comparisons, which allows us to assess the internal structure of the STN at the cellular level. Our results show a clear anatomical organization within the STN, and consistency across subjects. Our findings indicate that there are reoccurring patterns in the distribution of the individual immunocytochemical markers.

## Results

We obtained ten formalin-fixed tissue blocks containing the left STN from clinically non-demented donors via the Netherlands Brain Bank (www.brainbank.nl). Right STNs were used for neuropathological assessments and were, therefore, not available for our research. Clinicopathological data are presented in Table 1.

**Table 1:** Clinicopathological data

NBB#	Age (y)	Sex	PMD (h:m)	Fix (days)	*Cause of death*, clinical diagnosis
12-062	88	M	05:40	Nd	*Ischemic bowel rupture*, aortic stenosis, femoropopliteal bypass, hypercholesterolemia, cardio-renal syndrome, ischemic cardiomyopathy, atrial fibrillation, tauopathy^a^
12-082	101	F	05:10	Nd	*Cachexia*, cataract, TIA, mitral valve insufficiency, osteoporosis, coxarthrosis, kyphosis, decubitus, dehydration, AD Braak score 4C^a^
12-104	79	M	06:30	Nd	*Respiratory insufficiency*, type 2 diabetes, nephropathy
13-095	101	F	06:15	57	*Pneumonia*, cardiac failure, angina pectoris, cataract, hysterectomy, cholecystectomy, type 2 diabetes, coxarthrosis, spondylosis, conjunctivitis, COPD, bullous pemphigoid
14-037	101	F	07:27	57	*Renal insufficiency*, urinary tract infection, gastroenteritis, scoliosis, atrial fibrillation, cataract, prosthetic hip, AD Braak score 4C^a^
14-051	92	M	07:45	57	*Cardiac failure*, type 2 diabetes, polyneuropathy, decubitus, ascites, liver cirrhosis, cataract, COPD, prosthetic hips
14-069	73	M	04:25	56	*Pneumonia*, COPD, hypercholesterolemia, atrial fibrillation, aortofemoral bypass, PCTA, cataract, spondylosis, esophagitis, prostate carcinoma, hyperthyroidism, decubitus
15-033	93	M	07:40	59	*Cardiac failure*, aortic stenosis, cardiac decompensation, macular degeneration, basal cell carcinoma
15-035	73	M	08:00	56	*Pneumonia, cardiac failure*, pneumonia, myelodysplastic syndrome, fungal infection
15-055	72	F	06:50	55	*Respiratory insufficiency*, polymyalgia, polio, ovarium carcinoma, ileus, osteoporosis

*AD* Alzheimer’s disease, *Fix* fixation duration, *Nd* not determined, *PTCA* percutaneous transluminal coronary angioplasty, *PMD* postmortem delay, *TIA* transient ischemic attack, *y* years

^a^Determined postmortem

**Fig. 1:**
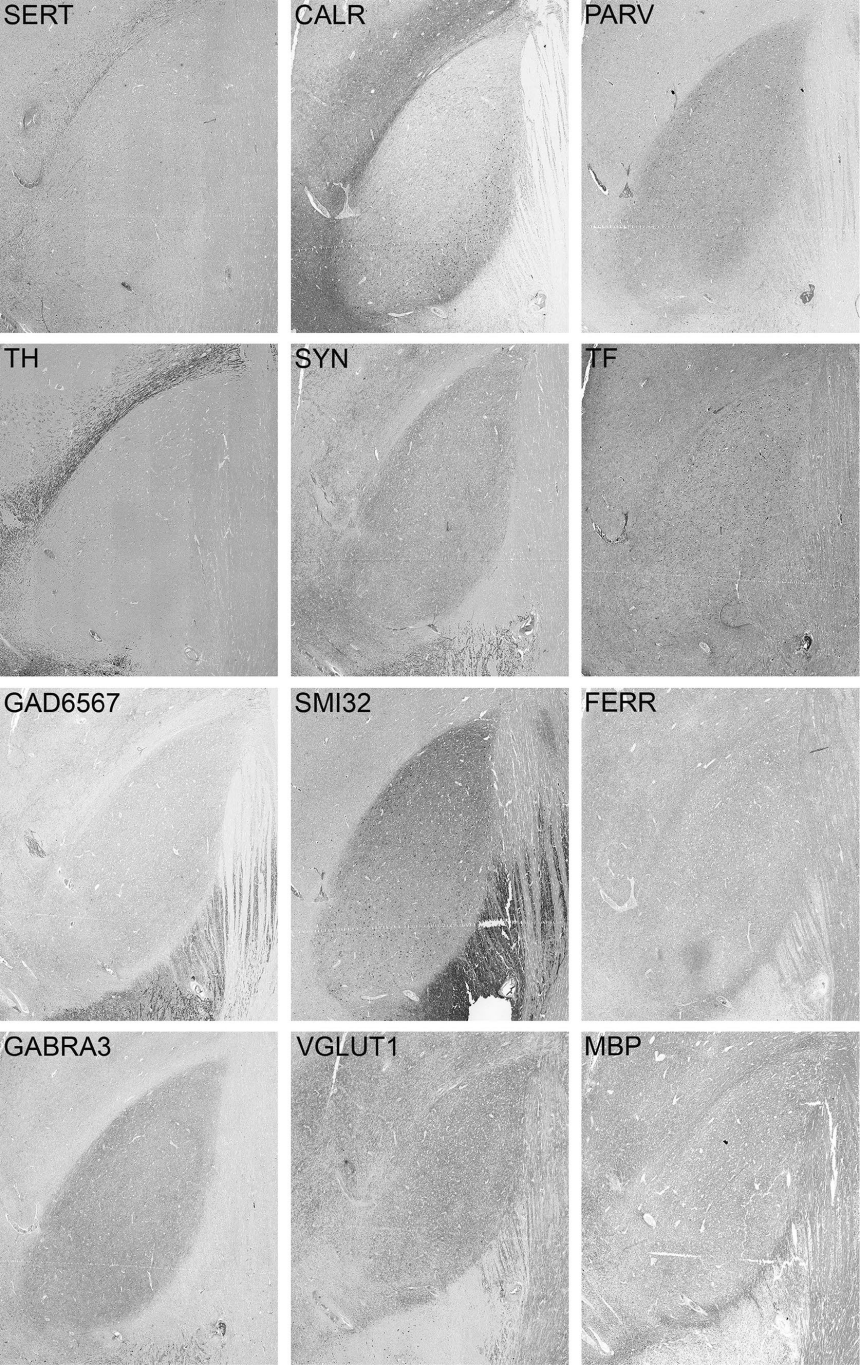
Example of immunoreactivity in specimen #14-051 for serotonin transporter (SERT), calretinin (CALR), parvalbumin (PARV), tyrosine hydroxylase (TH), synaptophysin (SYN), transferrin (TF), glutamic acid decarboxylase (GAD65/67), neurofilament H (SMI32), ferritin (FERR), GABA receptor subunit A3 (GABRA3), vesicular glutamate transporter 1 (VGLUT1), myelin basic protein (MBP)

Twelve primary antibodies were used for immunocytochemical studies of the STN. Antibody selection was based on the ability to label general cell features or major neurotransmitter systems, and reports on their expression in the STN (see “Methods”). Consecutive sections containing the STN were stained for (1) Neurofilament H (SMI-32), which showed clear labeling of the neuronal cell bodies. Additionally, weak fiber staining was present, and occasionally, long thin fibers were stained in the dorsolateral part of the STN; (2) synaptophysin (SYN), which showed punctate staining scattered throughout the nucleus, as well as neuronal somata surrounded by puncta. Additionally, the punctate staining extended beyond the dorsolateral tip of the nucleus in the shape of a cap. (3) Myelin basic protein (MBP) revealed clear labeling of myelin sheaths throughout the nucleus, (4) tyrosine hydroxylase (TH), which was observed in thick long, and thin punctate fibers. (5) Vesicular glutamate transporter 1 (VGLUT1) showed punctate fiber labeling. Increased density was present at the borders of the nucleus. (6) Glutamate decarboxylase 65/67 (GAD65/67) showed moderate fiber terminal staining; positive neurons were observed only occasionally. Additionally, presynaptic boutons were observed extending beyond the dorsolateral border of the STN, appearing as a cap on the dorsolateral tip of the nucleus. (7) GABA-A receptor subunit alpha 3 (GABRA3) showed predominant neuronal staining, in addition to punctate fiber staining. (8) Serotonin transporter (SERT), showed clear fiber labeling. (9) Parvalbumin (PARV) revealed clear labeling of cell bodies, as well as diffuse labeling of fibers; (10) calretinin (CALR) labeled both cell bodies and fibers. (11) Transferrin (TF) labeling was present in oligodendrocytes, as well as numerous blood vessels. The oligodendrocytes displayed a clear rounded shape and cytoplasmic staining. Positive neurons were detected frequently while few fibers were also present. Transferrin staining showed substantial background staining, which fits with transferrin labeling in the extracellular matrix. (12) Ferritin (FERR) staining revealed numerous positive oligodendrocytes, as well as weaker punctate staining. For illustrations, see Fig. 1 and supplementary material 1.

**Fig. 2:**
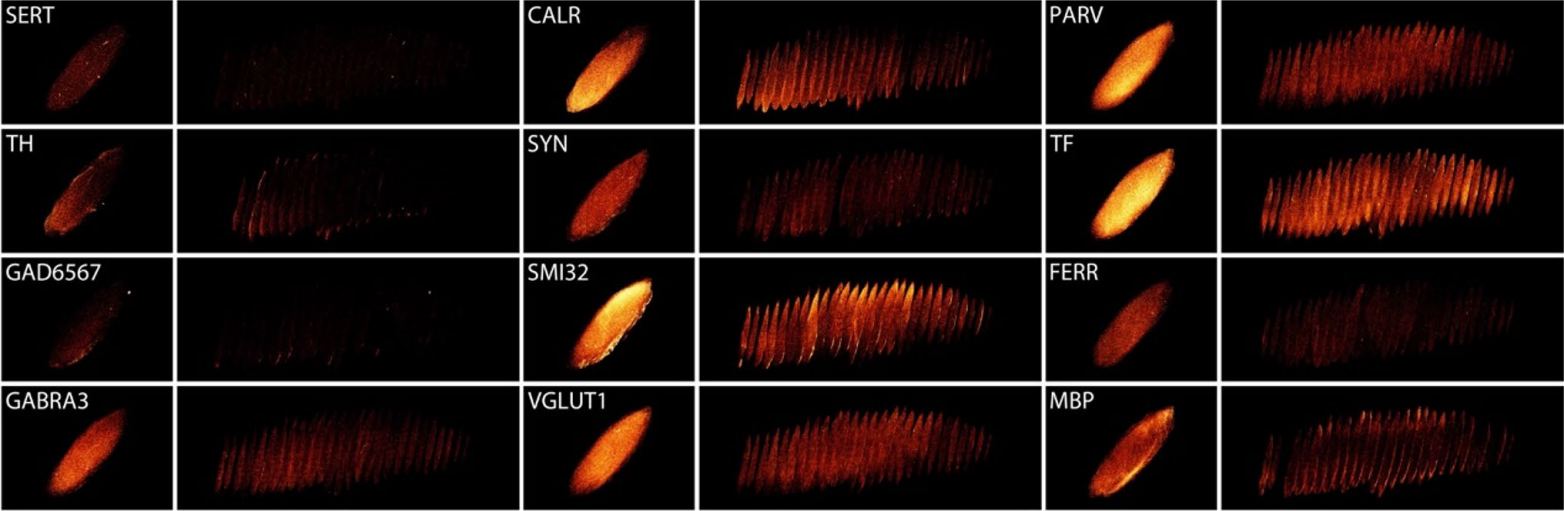
Example of a single STN (#15-033). Immunoreactivity is presented as maximum intensity Z-stacks, as well as consecutive sections for serotonin transporter (SERT), calretinin (CALR), parvalbumin (PARV), tyrosine hydroxylase (TH), synaptophysin (SYN), transferrin (TF), glutamic acid decarboxylase (GAD65/67), neurofilament H (SMI32), ferritin (FERR), GABA receptor subunit A3 (GABRA3), vesicular glutamate transporter 1 (VGLUT1), myelin basic protein (MBP). Note that the panels reflect immunoreactivity, not protein copies. Therefore, differences across markers cannot be interpreted as differences in protein expression levels

The antibodies include markers for the principal excitatory glutamatergic system, the principle inhibitory GABA-ergic system as well as other major neurotransmitter systems such as dopamine and serotonin. A more extensive overview of the characteristics of the antibodies is presented in “Methods”. Immunoreactivity was visually inspected and present for all proteins in all tested tissue specimens, and staining intensity showed substantial interindividual variation, which is in line with previous publications (Alkemade et al. 2012; Borgers et al. 2014). Digital images were created and shape information was used to perform linear transformations for registration to the corresponding block face images. Additionally, manual outlines of the STN were created by two independent raters on PARV and SMI32 sections to define the location and outline of each individual STN. These STN outlines and the thresholded staining results allowed the reconstruction of 3D densitometric data (arbitrary units) into block face space in seven tissue specimens as illustrated in Fig. 2. For three other blocks, 3D reconstructions were not further analyzed, due to poor quality as a result of distortions and tissue damage. Visual inspection showed protein marker expression throughout the entire STN, for each marker, with clear local intensity differences for each marker.

To investigate the consistency of the immunoreactivity patterns across specimens, each individual 3D reconstruction of an individual staining pattern was rasterized into 3 × 3 × 3=27 sectors of equal volume along the rostrocaudal axis, and the dorsolateral–ventromedial, and its orthogonal axis. For each sector and protein marker, the mean staining intensity was tested against the mean of the sectors. For 10 out of 12 markers, a consistent inhomogeneous immunoreactivity pattern was identified in one or more sector using one-sample *t* tests, thresholded at a false discovery rate of *q* < 0.05.

We subsequently investigated the immunoreactivity patterns in more detail, and tested whether they were best described using a gradient model or using a tripartite model. We rasterized the STN in 1000 voxels in the dimensions identified with the PCA analysis described above (10 voxels in each direction). We then fit four generalized linear models (GLMs) with negative binomially distributed errors for each specimen and protein marker. These GLMs describe the expression in each voxel as a function of location in space along the PCA dimensions. Model A assumes no change across space (homogenous immunoreactivity):

$$\left[ {{\text{EQ}} 1} \right] y = \exp \{ \lambda_{0} \} + \epsilon ,\;\,\epsilon \sim {\text{negative}}\;{\text{binomial}}\left( \alpha \right)$$

in which *y* is the observed immunoreactivity, $\lambda_{0}$ an intercept, and *α* the gamma distribution parameter that is assumed to underlie the rate distribution of a Poisson process. Model B assumes that the change in expression is linear across space:

$$y = \exp \left\{ {\lambda_{0} + \lambda_{1} x_{1} + \lambda_{2} x_{2} + \lambda_{3} x_{3} } \right\} + \epsilon,$$

$$\epsilon \sim {\text{negative}}\;{\text{binomial}}\left( \alpha \right)$$

where *x*_1_–*x*_3_ are the voxels’ location along the rostrocaudal, the dorsomedial–ventrolateral, and its orthogonal axes, respectively, and $\lambda_{1 - 3}$ the corresponding weights. Model C assumes three subdivisions separated by abrupt boundaries:

$$\beta_{2}^{\prime } = \left( {1 - \beta_{1} } \right),$$

$$\beta_{3}^{\prime } = \left( {1 - \beta_{1} - \beta_{2}^{\prime } } \right),$$

$$p = \beta_{1} x_{1} + \beta_{2}^{\prime } x_{2} + \beta_{3}^{\prime } x_{3},$$

$$d_{1} \left\{ {\begin{array}{*{20}l} 1 \hfill & {\quad {\text{if}}\;p < \tau_{1} *\left\| p \right\|} \hfill \\ 0 \hfill & {\quad {\text{otherwise,}}} \hfill \\ \end{array} }, \right.$$

$$d_{2} \left\{ {\begin{array}{*{20}l} 1 \hfill & {\quad {\text{if}}\;p > \tau_{2} *\left\| p \right\|} \hfill \\ 0 \hfill & {\quad {\text{otherwise,}}} \hfill \\ \end{array} }, \right.$$

$$y = \exp \left\{ {\lambda_{0} + \lambda_{1} d_{1} + \lambda_{2} d_{2} } \right\} + \epsilon , \;\epsilon \sim {\text{negative}}\;{\text{binomial}}\left( \alpha \right),$$

where each voxel location is projected onto a new axis *p*, which defines the axis along which the borders $\tau_{1 - 2}$ are located. Finally, Model D assumes a gradient of non-linear changes:

$$\beta_{2}^{\prime } = \left( {1 - \beta_{1} } \right),$$

$$\beta_{3}^{\prime } = \left( {1 - \beta_{1} - \beta_{2}^{\prime } } \right),$$

$$p = \beta_{1} x_{1} + \beta_{2}^{\prime } x_{2} + \beta_{3}^{\prime } x_{3} ,$$

$$\begin{aligned} y & = { \exp }\left\{ {\lambda_{0} + \frac{{\lambda_{1} }}{{1 + \exp \left\{ { - \kappa \left( {\left( {\tau_{1} *\left\| p \right\|} \right) - p} \right)} \right\}}} + \frac{{\lambda_{2} }}{{1 + \exp \left\{ { - \kappa \left( {\left( {\tau_{2} *\left\| p \right\|} \right)) - p} \right)} \right\}}}} \right\} \\ & \quad + \epsilon , \;\epsilon \sim {\text{negative}}\;{\text{binomial}}\left( \alpha \right), \\ \end{aligned}$$

such that changes across space are described by a sigmoidal function with smoothness parameter $\kappa$. Variables used are *y* = stain intensity, *x*1, 2, 3 = voxel locations along the three axes, *p* = projection axis, ||*p*|| = length of projection axis, *d*1/*d*2 = dummy variables.

The Bayesian Information Criterion (Schwarz 1978; Wagenmakers and Farrell 2004) was used to compare the quality of fit of the four GLMs. The BIC provides a measure of the quality of model fit penalized for model complexity to provide a quantification of parsimony. The results of the model comparison are shown in Table 2. Model B (linear gradient) provided the best fit in 61.9% of the specimen/protein marker combinations, and is the overall preferred model as evidenced by the highest mean weighted BIC. Non-linear gradient Model D was preferred in 29.76% of the cases, and the tripartite model provides the best description of the data for the remaining 8.33% of the specimen/protein marker combinations (Table 2).

**Table 2:** Preferred model specimens and protein marker defined as the model with the lowest BIC

NBB#	CALR	FER	GABRA3	GAD6567	MBP	PARV	SERT	SMI32	SYN	TH	TRANSF	VGLUT1	Overall
13-095	B	B	D	B	D	D	C	B	D	B	D	B	B
14-037	B	B	B	B	B	B	B	B	D	D	B	D	B
14-051	B	D	B	B	D	D	B	B	D	B	D	B	B
14-069	B	B	B	B	D	B	B	C	C	C	C	D	B
15-033	B	B	B	B	B	B	B	B	B	B	B	B	B
15-035	B	C	D	B	B	D	B	D	B	B	B	C	B
15-055	B	B	D	B	D	D	B	D	D	D	B	D	D
Across specimens	B	B	B	B	D	D	B	B	D	B	B	B	B

Model A represents homogenous immunoreactivity across the nucleus; Model B represents a linear gradient model; Model C assumes three subdivisions; Model D assumes a gradient of non-linear changes

**Fig. 3:**
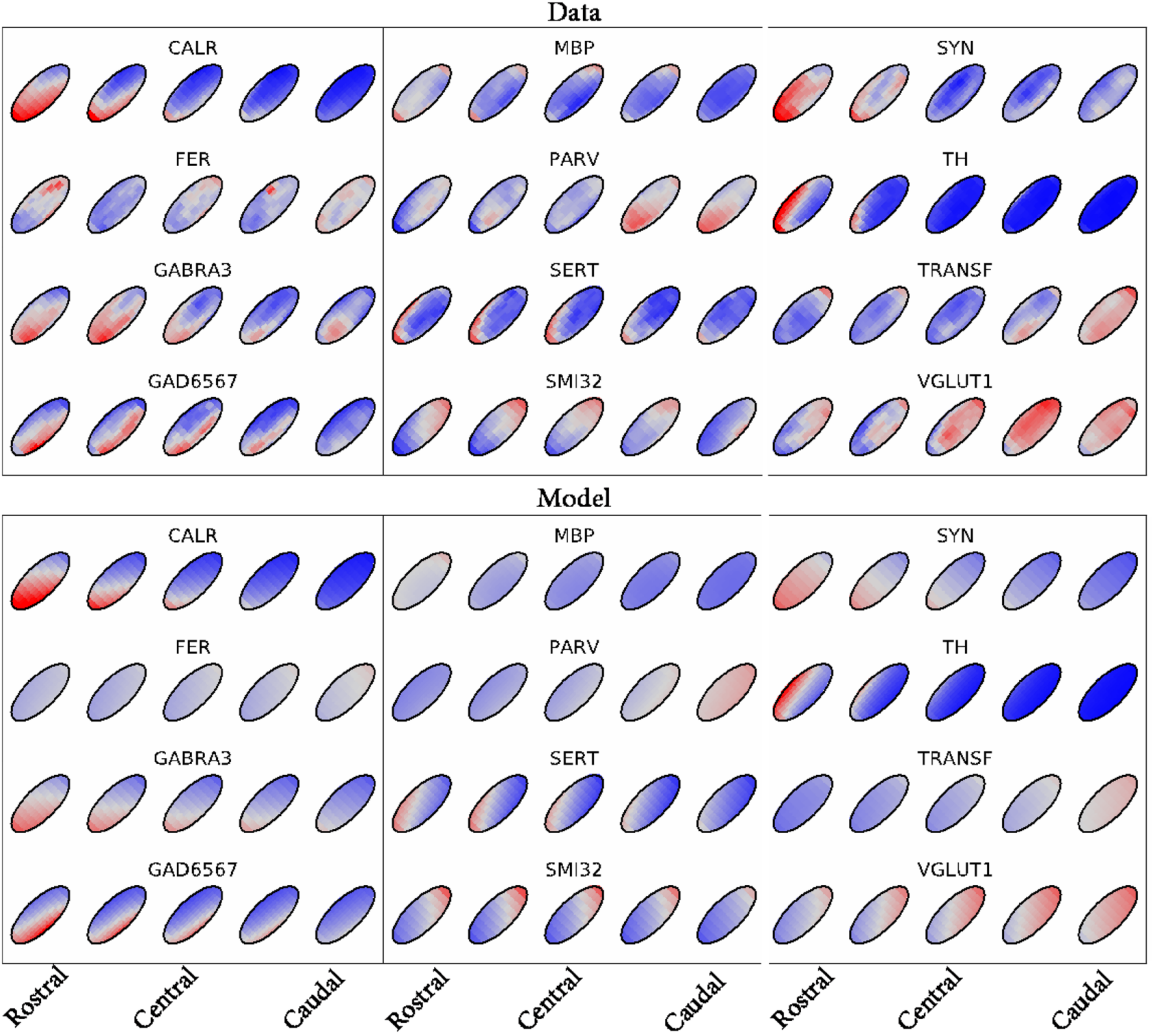
Quality of fit for Model B (linear gradient) over the rostrocaudal axis of the STN. Top panels show the data, and bottom panels show the model. Colors represent overall mean normalized intensity for the data, and the model. Relative changes in immunoreactivity are indicated in colors, ranging from relatively low (blue) to high (red) expression levels. Note that protein marker expression was present throughout the entire STN, for each marker, with clear local intensity differences within markers

Figure 3 illustrates the overall quality of the fit of Model B. For each specimen, individual immunoreactivity data and model predictions were normalized to mean 0 and standard deviation 1. Model B, which described a linear gradient, was able to capture the global patterns in expression across space best for protein markers CALR, GABRA3, GAD6567, SERT, SMI32, SYN, TH, and VGLUT1. MBP, FER, PARV, and TRANSF show less consistency across specimens and/or highly non-linear patterns across space (e.g., local spheres of expression, c.f. PARV).

## Discussion

In the present study, we provide spatial detail and 3D information that allow us to move beyond descriptive accounts of the anatomy of the human STN. The results confirm that the STN is not a homogenous nucleus, nor does the organization of the STN support of the existence of subdivisions as defined by clustered neuronal populations with limited border zones.

An inherent limitation of postmortem studies is a bias towards old age, as well as strong variation in antemortem disease and cause of death. Since we collected the tissue from clinically non-demented controls in a prospective fashion, we did not select donors with comparable disease state, as described previously (Alkemade et al. 2003, 2005b). By chance, we included three female centenarians in our studies. In view of these limitations, we cannot draw any meaningful conclusions on potential effects of factors such as age, sex or disease state. We would like to stress that immunoreactivity is determined by protein expression, as well as the staining procedure. Therefore, no meaningful conclusions can be drawn from comparisons across antibodies within the current studies or from comparisons to reports on the abundance of immunoreactivity across species in literature. All comparisons described here are, therefore, performed exclusively within and not across the individual markers. Our data are in agreement with earlier results on the human and nonhuman primate STN (Nauta and Cole 1978; Mori et al. 1985; Kultas-Ilinsky et al. 1998; Augood et al. 1999, 2000; Hedreen 1999; Charara et al. 2000; Hurd et al. 2001; Kuwajima et al. 2004; Levesque and Parent 2005; Aubert et al. 2007; Parent et al. 2011), and allow us to speculate further on the existence of functional subdivisions in the STN. Distinct PARV and CALR distributions suggest local differences in intracellular calcium dynamics in the STN, and highest immunoreactivity indicative of GABA-ergic signaling was located in the ventromedial part of the anterior half of the nucleus. This did not coincide with the most intense glutamatergic immunoreactivity, which was located in the dorsocaudal extent of the STN. It is important to note that both markers for GABA- and glutamatergic signaling were expressed throughout the STN. The variability in intensity distributions between the 12 individual markers points towards a complex STN organization, indicating that the tripartite hypothesis of the STN represents an oversimplification of its internal structure.

Our statistical modeling reveals consistent gradients in the immunoreactivity patterns throughout the human STN. Interpreting these findings within the framework of functional segregation, the spatial segregation of individual cell populations within the STN during development appears to occur only to some extent at best, resulting in incomplete functional segregation within the STN. Small nonhuman primate studies show that neurons located in the dorsolateral part of the STN are connected to the globus pallidus externa, whereas neurons connected to the globus pallidus interna, substantia nigra pars reticulata, and caudate nucleus are large, although not exclusively confined to the ventromedial parts of the STN (Nauta and Cole 1978; Smith et al. 1990). In addition, neurons projecting to the ventral globus pallidus are located in the medial STN (Nauta and Cole 1978; Smith et al. 1990). Thorough tracing studies in nonhuman primates have revealed a clear topological organization of connectivity in the STN, but tracing techniques inherently lead to an underestimation of the quantity and distribution of projections in the STN (Alkemade 2013; Haynes and Haber 2013).

In vivo imaging studies, as well as clinical observations, provide some support for zonation within the STN [see (Alkemade et al. 2015) for review], although caution should be applied when interpreting these data, since the limited anatomical detail does not allow reliable assessment of the level of anatomical segregation between potential functional subdivisions. Here, we have studied that the internal structure of the STN within the framework of functional segregation, and the complex neuroanatomical structure, including the strong overlap of the distribution patterns of the immunocytochemical markers indicates that there is little evidence for anatomical segregation. We, therefore, conclude that the distribution of immunoreactivity as described here do not align with proposed functional subdivisions (Keuken et al. 2012; Alkemade and Forstmann 2014), and is in support of limited local function specialization. It is difficult to predict the importance of the observed topographical variations for the mechanism underlying DBS. STN electrodes inserted to treat a number of motor disorders are aimed at the dorsolateral part of the STN (Greenhouse et al. 2013). In line with the results of Haynes and Haber (Haynes and Haber 2013), who showed significant overlap in projection patterns within the primate STN, we found that none of the tested markers was confined to the dorsolateral STN, and we found no evidence for anatomical borders with clear subdivisions.

## Methods

### Tissue processing

All brain tissues were collected within a 4-year period with a < 8-h postmortem interval before brain autopsy. Tissues were obtained in accordance with BrainNet Europe’s Code of Conduct for brain banking (Klioueva et al. 2015). Dissection of the STN was performed at the time of autopsy, and all tissue specimens were subjected to the same tissue processing procedure. Tissue blocks were dehydrated and paraffin embedded. Block face imaging was performed while cutting serial coronal sections (6 µm) and images were realigned for reconstruction purposes. Sections were systematically sampled with 300-µm intervals, stained, and digitally imaged to produce a 3D reconstruction of the staining profiles in block face space.

Tissue was fixated for approximately 8 weeks in 10% formalin (10× V/V). After initial formalin fixation, tissues were transferred to phosphate-buffered saline [PBS (pH ~ 6.6–7.0): 145 mM NaCl, 9 mM disodium phosphate (Na_2_HPO_4_ cat. no. 71640, Sigma-Aldrich, St. Louis, MO, USA), 2 mM sodium phosphate (NaH_2_PO_4_·H_2_O, cat. no. S9638, Sigma-Aldrich, St. Louis, MO, USA)] containing 0.02% sodium azide to prevent fungal growth until further processing. Following dehydration, tissues were embedded in paraffin, after which 6-µm-thick serial coronal sections were cut covering the rostrocaudal axis of the STN. While processing, block face imaging was performed (at an interval of 50 sections), which provides an intermediate step crucial for 3D reconstructions. This was done using a camera mounted in front of the tissue blocks.

At the block face imaging level, sections were mounted on Menzel-Gläser Superfrost plus object slides (Cat. no. J1800AMNZ, Thermo Scientific, Braunschweig, Germany) and stained with thionine (Thionine acetate: cat. no. 1.15929.0025, Merck, Darmstadt, Germany) for anatomical orientation. Sections were stained at 300-µm intervals, which allowed us to identify the borders of the STN using a light microscope. At each level, consecutive sections containing the STN were mounted for immunocytochemical staining of neurofilament H (SMI-32), synaptophysin (SYN), tyrosine hydroxylase (TH), vesicular glutamate transporter 1 (VGLUT1), glutamate decarboxylase 65/67 (GAD65/67), GABA-A receptor subunit alpha 3 (GABRA3), serotonin transporter (SERT), parvalbumin (PARV), calretinin (CALR), transferrin (TRANSF), and ferritin (FERR) (see Table 3).

**Table 3:** Primary antibody characteristics

Primary Ab	Dilution	Protein	Function	Source	Specificity
Pilot studies					
NPY	1:1,000	Neuropeptide Y	Neurotransmitter, affects cortical excitability, stress response, food intake, circadian rhythms, and cardiovascular function	Niepke, NIN	IEF, preadsorptions, omission primary Ab, ICC (van der Beek et al. 1992)
CRH	1:100,000	Corticotropin-releasing hormone	Peptide hormone/neurotransmitter involved in the stress response	PFU83, Free University of Amsterdam	Preadsorptions, preimmune serum testing, omission primary Ab, ICC (Raadsheer et al. 1993; Erkut et al. 1995)
ORXA	1:20,000	Orexin A	Peptide hormone involved in sleep regulation	H003-30, Phoenix Pharmaceuticals	Preadsorptions, dot blots, ICC (Fronczek et al. 2005)
VIP	1:1,000	Vasoactive intestinal peptide	Peptide hormone involved in circadian rhythmicity	Viper, NIN	IEF, preadsorptions, omission primary Ab, ICC (van der Beek et al. 1992)
Aromatase	1:1,500	Aromatase	Enzyme involved in estrogen synthesis	Narom, NIN	ICC (van der Beek et al. 1992)
ChAT	1:200	Choline acetyl transferase	Enzyme involved in acetylcholine synthesis		ICC (Dubelaar et al. 2004)
Distribution studies					
SMI-32	1:2,000	Neurofilament H	Major cytoskeletal component	SMI-32, Covance	ICC, WB (Bar-Peled et al. 1997; Fernyhough et al. 1999; Brownlees et al. 2000; Gveric et al. 2001; Van den Haute et al. 2001; Weigum et al. 2003; Van der Gucht et al. 2003; Veeranna et al. 2004; Mahad et al. 2009; Jablonska et al. 2012; Sareen et al. 2012)
SYN	1:250	Synaptophysin	Major synaptic vesicle protein	A0010, DAKO	ICC, WB, ELISA (Sager et al. 2000; Hara et al. 2008; van Vliet et al. 2009; Gottschall et al. 2010; Eftekhari and Edvinsson 2011; Purushothuman et al. 2013)
TH	1:1,500	Tyrosine hydroxylase	Rate-limiting enzyme in dopamine production (Nagatsu et al. 1964)	MAb318, Millipore	ICC, WB (Perez et al. 2002; Kanaan et al. 2007; Morrow et al. 2007; Mastroberardino et al. 2009; Dobi et al. 2010; Thomsen et al. 2010; Addis et al. 2011; Mulcahy et al. 2012; Rothmond et al. 2012)
VGLUT1	1:10,000	Vesicular glutamate transporter 1	Sodium dependent phosphate transporter, glutamate signaling (Takamori et al. 2000)	135302, Synaptic systems	ICC, WB (Kirvell et al. 2006; Zhou et al. 2007; Ribic et al. 2010; Zander et al. 2010; Larsson et al. 2012; Kempf et al. 2013; Nair et al. 2013; Sun et al. 2013)
GAD65/67	1:300	Glutamate decarboxylase 65/67	Enzyme that catalyzes glutamate to GABA conversion	Ab1511, Millipore	ICC, WB (Mori et al. 1985; Hedou et al. 2000)
GABRA3	1:250	GABA-A receptor subunit alpha 3	Receptor subunit	AGA-003, Alomone Labs	ICC, WB (Caspary et al. 2013; Zhou et al. 2013; Park et al. 2013; Seo and Leitch 2014)
SERT	1:5,000	Serotonin transporter	Determines serotonin availability in the synaptic cleft (Bengel et al. 1998)	MAb5618, Millipore	ICC, WB (Bauman et al. 2000; Ramsey and DeFelice 2002; Serafeim et al. 2002; Henry et al. 2003; Borgers et al. 2014)
PARV	1:2,500	Parvalbumin	Calcium-binding protein	195004, Synaptic systems	ICC (Andrioli et al. 2007; Gross et al. 2011; Mallet et al. 2012; Huang et al. 2013; Lee et al. 2013)
CALR	1:450	Calretinin	Calcium binding protein	6B3, Swant	ICC, WB (Schiffmann et al. 1999; Lavenex et al. 2009; Fuentealba et al. 2010; Lancaster et al. 2013)
TRANSF	1:4,000	Transferrin	Iron-binding glycoprotein	Ab9538, Abcam	ICC, WB (Zawadzka et al. 2010)
FERR	1:1,500	Ferritin	Iron-binding protein expressed in oligodendrocytes (Vymazal et al. 2000; Leitner and Connor 2012)	sc-14416, Santa Cruz	ICC, WB (Saunders et al. 2007; Vidal et al. 2008; Sengupta et al. 2009; Li et al. 2009; Duan et al. 2009)

*Ab* antibody, *ELISA* enzyme-linked immunosorbent assay, *ICC* immunocytochemistry, *IEF* isoelectric focusing, *WB* Western Blotting

Each section was stained using a single antibody. In short, paraffin was cleared from the slides using xylene and tissues were rehydrated using a graded series of alcohols. After rinsing in distilled water, antigen retrieval was performed using heat treatment (Shi et al. 1997), and pre-incubation was performed if appropriate. Subsequent incubation in the primary antibody was performed overnight in a humidified chamber in Supermix [(SUMI): TBS containing 0.25% gelatin (cat. no. 1.04078.1000, Merck, Darmstadt, Germany) and 0.5% Triton X-100 (cat.no. X100, Sigma-Aldrich, St. Louis, MO, USA)]. After rinsing in Tris-buffered saline [(TBS): 150 mM NaCl (cat. no. 1.06404.1000, Merck, Darmstadt, Germany), 50 mM Tris–HCl, pH 7.6 (Trizma cat. no. T1503, Sigma-Aldrich, St. Louis, MO, USA)], sections were incubated in an appropriate biotinylated secondary antibody (Vector laboratories Inc., Burlingame, CA, USA), followed by incubation in avidin-biotinylated complex (VECTASTAIN ABC Kit: cat. no. PK-6100, Vector laboratories Inc., Burlingame, CA, USA) and visualization of the staining using diaminobenzidine amplified with nickel ammonium sulphate [DAB: cat. no. D5637, Sigma-Aldrich, St. Louis, MO, USA; Ammonium nickel (II) sulfate: BDH Chemicals, UK] as a chromogen resulting in an intense purple precipitate as described previously (Alkemade et al. 2005a, c, 2012; Borgers et al. 2013). Sections were cover slipped using Entellan (Cat. no. 1.0791.0500, Merck, Darmstadt, Germany).

### Image processing

Block face images were restacked using image J (1.48 V), Stackreg (Thévenaz et al. 1998). Tissue borders were manually outlined in the block face image since the more caudal tissue was visible in the paraffin resulting in limited contrast that did not allow reliable automatic parcellation of the tissue in the field of view.

All stained sections were digitally imaged using a slide scanner (Ventana iScan HT, Roche). The appropriate image was selected and extracted from the BigTiff format, and the image was adjusted to allow registration of the protein markers to the appropriate block face images. Image conversion was performed to exclude contrast in the tissue and to allow registration based on shape of the tissue. This was done because the contrast information obtained from the block face image was substantially different from that obtained from staining. Images were registered using a scaled rotation, followed by an affine transformation. Transformation matrices were saved. All transformations were visually inspected, and if the results were unsatisfactory, as evidenced by clear jumps of the sections within the reconstructed STN structure, images were registered using a rigid body transformation followed by an affine registration. If registration results were still deemed to be insufficient, images were discarded. As a result, overall registration of #12-062, 12-082, and 12-104 was judged insufficient, and these specimens were excluded from further analyses.

### Thresholding of the staining

A histogram-based thresholding procedure was applied to remove background signal from the immunocytochemistry procedure on the red channel of the stained images in ImageJ, by creating a RGB-stack, followed by the default thresholding procedure implemented in ImageJ, similar to previous reports (Alkemade et al. 2012; Borgers et al. 2013; Ten Kulve et al. 2016). Threshold settings were determined experimentally [CALR (0, 95), FER (0, 127), GABRA (0, 134), GAD6567 (51, 112), MBP (0, 130), PARV (41, 133), SERT (0, 165), SMI (0, 156), SYN (0, 162), TRANSF (0, 110), TH (0, 155), VGLUT1 (0, 140)]. The thresholded images were converted using the binary mask functions to which subsequently the scaled rotation and affine transformation matrices, and, if appropriate, the rigid body and affine transformation matrices were applied (see Fig. 2 for an example). Two independent raters delineated the STN over the entire rostrocaudal axis of the STN using PARV and SMI32 images. The area included in the STN in a minimum of three masks was used for further analyses.

### Quantitative analyses of immunoreactivity

Immunocytochemical were brought to individual block face space for each specimen. These images had a resolution of 0.014 mm isotropic in the cutting plane, and 0.3 mm between adjacent slices. For the immunocytochemical images, the thresholded and transformed images constructed using imageJ were analyzed. These images were smoothed with a Gaussian kernel with a full-width half maximum (FWHM) of 0.3 mm (Szeliski 2010; de Hollander et al. 2014). This smoothing procedure was performed to (1) increase signal-to-noise ratio and (2) focus the analyses on topological patterns in the order of 0.1–1 mm, rather than patterns in structures much smaller than the STN. To ensure that image intensities outside the STN mask were not included in the analyses, the smoothing kernel was truncated outside the STN mask and normalized.

### Consistency of immunoreactivity patterns

To analyze the data of seven different STN specimens in a common space, they were rasterized in 27 sectors. Sectors were created by defining and dividing three axes in the STN into three equal parts. The first axis was defined by the rostrocaudal cutting plane. The two other axes were defined by a principal component analysis (Bishop 2006) on the 2D coordinates over all slices. The resulting axes were visually inspected and were consistently identified as a main dorsolateral → ventromedial axis and a mediodorsal → ventromedial axis.

For each individual specimen, sectors were subjected to PCA analyses to test whether specific sectors showed altered immunoreactivity. For each specimen and antibody, the 27 sectors were demeaned and standardized, setting their mean at 0 and their standard deviation at 1. Subsequently, per sector, a one-sample *t* test was performed over the 7 STN specimens. Results underwent a false discovery rate correction to account for multiple comparisons. Significant *t* values indicated altered local expression levels.

STN specimens were then rasterized into 1000 sectors. The immunoreactivity in the identified sectors was entered as a dependent variable in a set of general linear models (GLMs). As an error function, the negative binomial was used to account for the observed overdispersion. The negative binomial describes a Poisson variable with a rate that is gamma distributed with parameter *α*.

Models were fit using maximum likelihood estimation. Since maximum likelihood estimation is susceptible to outlying data points, sectors with extremely high intensity (defined here as five times the interquartile range above the median after a log-transformation) were excluded from these analyses (0.07% of all data points). Optimization was performed using differential evolution (Storn and Price 1997) with population sizes set to 20 times the number of parameters in a model, and up to 5000 iterations. Subsequently, the parameters were refined using least-squares optimization. Both optimization algorithms were implemented in Python library *Scipy* [version 1.2.0 (Jones E, Oliphant E, Peterson P)**]**. Parameter optimization bounds were set between [− 15, 200] for $\lambda_{0}$ (all models) and $\lambda_{1 - 2}$ (Models C–D); [− 2, 2] for $\lambda_{1 - 3}$ (Model B); [0, 1] for $\beta_{1 - 2}$ (Models 3–4); [0.2, 0.6] for $\tau_{1}$ and [0.4, 0.8] for $\tau_{1}$; [1, 1×10^20^] for $\kappa$ (Model D) and *α* (all models). Parameters $\tau_{1 - 2}$ were constrained to ensure that each fit subdivision spans at least 20% of the length of the projection axis *p*, to prevent the optimization routine from identifying very small, anatomically implausible subdivisions.

The Bayesian information criterion [BIC (Schwarz 1978; Wagenmakers and Farrell 2004)] was used for model comparison. It is defined as ${\text{BIC}} = - 2\log \left( L \right) - k\log \left( n \right)$, where *L* is the likelihood of the data under the model, *k* the number of parameters of the model, and *n* the number of data points. Lower BIC values indicate a more parsimonious trade-off between quality of fit and model complexity. To compare BICs across protein markers and specimens, the weighted BIC (wBIC) of each model *i* was used:

$$w_{i} {\text{BIC}} = \frac{{{\text{e}}^{{ - \frac{1}{2}\Delta_{i} \left( {\text{BIC}} \right)}} }}{{\mathop \sum \nolimits_{k = 1}^{K} {\text{e}}^{{ - \frac{1}{2}\Delta_{k} \left( {\text{BIC}} \right)}} }},$$

where $\Delta_{i} \left( {\text{BIC}} \right) = {\text{BIC}}_{i} - \hbox{min} \left( {\text{BIC}} \right)$. The wBIC values can be interpreted as the probability a model is the data-generating model under the assumption that the data-generating model is among the models under consideration (Wagenmakers and Farrell 2004). Higher wBIC values thus indicate more evidence for each model.

### Data and computer code availability

The 10 × 10 × 10 grid data corresponding to the 1000 sectors that were analyzed are available via https://figshare.com/s/aee5a09da245058504a9.

Computer code is available via: https://github.com/StevenM1/histochemical_mri_stn and https://github.com/StevenM1/pystain.

## Electronic supplementary material

Below is the link to the electronic supplementary material.

Supplementary material 1 (PDF 20146 kb)
