# Supplementary material for: The functional microscopic neuroanatomy of the human subthalamic nucleus
Source: Brain Struct Funct. 2019 Sep 28;224(9):3213–27. doi: 10.1007/s00429-019-01960-3 (PMC6875153; doi:10.1007/s00429-019-01960-3)

Supplementary data for Brain Structure and Function:

The Functional Microscopic Neuroanatomy of the Human Subthalamic Nucleus

Anneke Alkemade†[,1], Gilles de Hollander†[,2,3], Steven Miletic†[,1], Max C Keuken[1], Rawien Balesar[1], Onno de Boer[4], Dick F Swaab[5], Birte U Forstmann[1,*]

Affiliations:

[1]Integrative Model-Based Neuroscience Research Unit of the University of Amsterdam, Amsterdam, The Netherlands.

[2]Laboratory for Social and Neural Systems Research (SNS-Lab), Department of Economics, University of Zurich, Zurich, Switzerland.

[3]Spinoza Centre for Neuroimaging, Royal Academy of Sciences, Amsterdam, the Netherlands

[4]Department of Pathology, location AMC of the Amsterdam UMC, Amsterdam, The Netherlands.

[5]Department of Neuropsychiatric Disorders, The Netherlands Institute for Neuroscience, an Institute of the Royal Netherlands Academy of Arts and Sciences, Amsterdam, The Netherlands.

†Equal contribution

*Corresponding author:

Prof Dr Birte U Forstmann

Integrative Model-based Cognitive Neuroscience (IMCN) Research Unit

University of Amsterdam

Nieuwe Achtergracht 129B | Room G3.06

PO Box 15926 | 1001 NK Amsterdam

buforstmann@gmail.com

Supplementary Figure 1: High power magnitifications of immunoreactivity in specimen #14-051 for serotonin transporter (SERT), calretinin (CALR), parvalbumin (PARV), tyrosine hydroxylase (TH), synaptophysin (SYN), transferrin (TF), glutamic acid decarboxylase (GAD65/67), neurofilament H (SMI32), ferritin (FERR), GABA receptor subunit A3 (GABRA3), vesicular glutamate transporter 1 (VGLUT1), myelin basic protein (MBP).

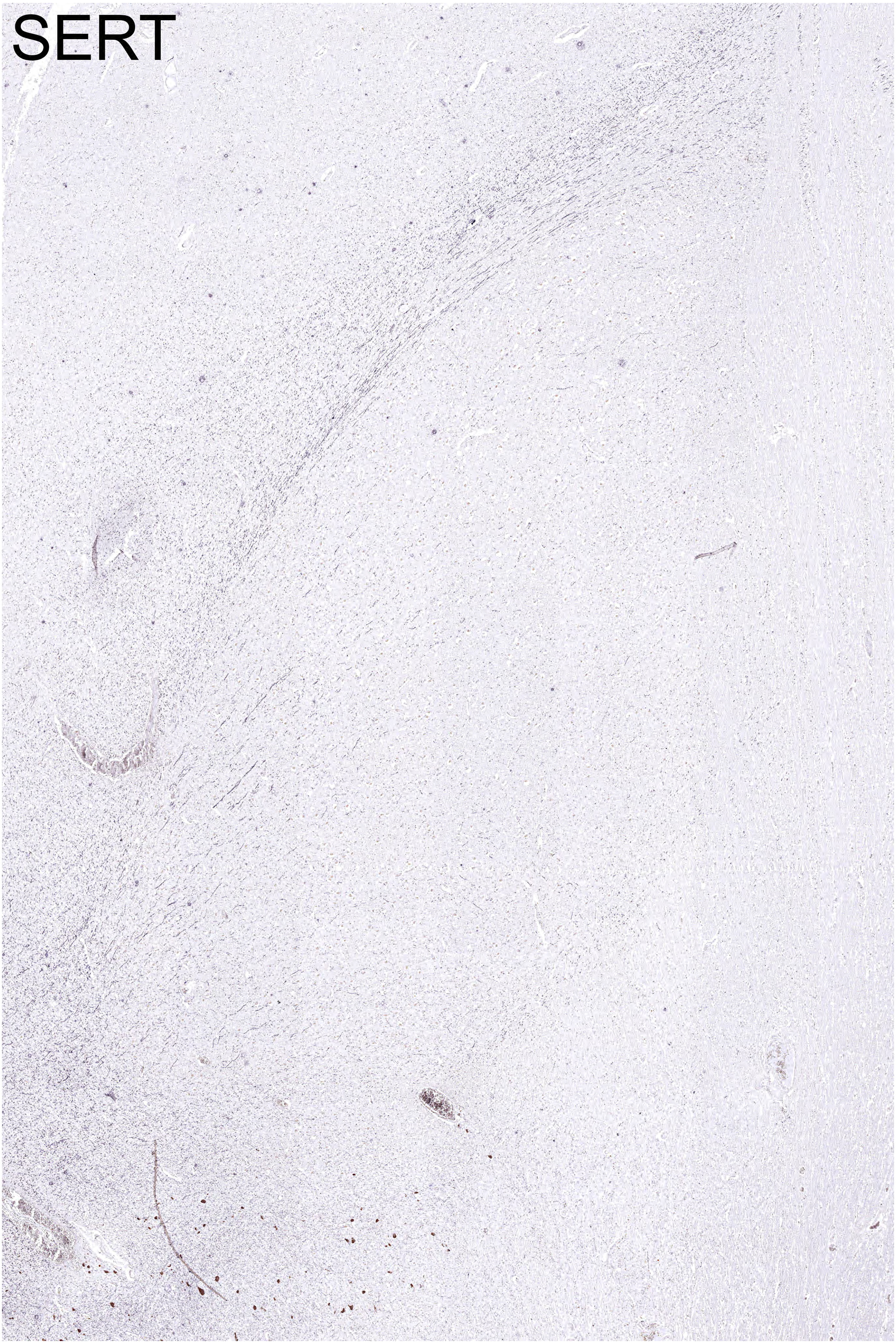
SERT

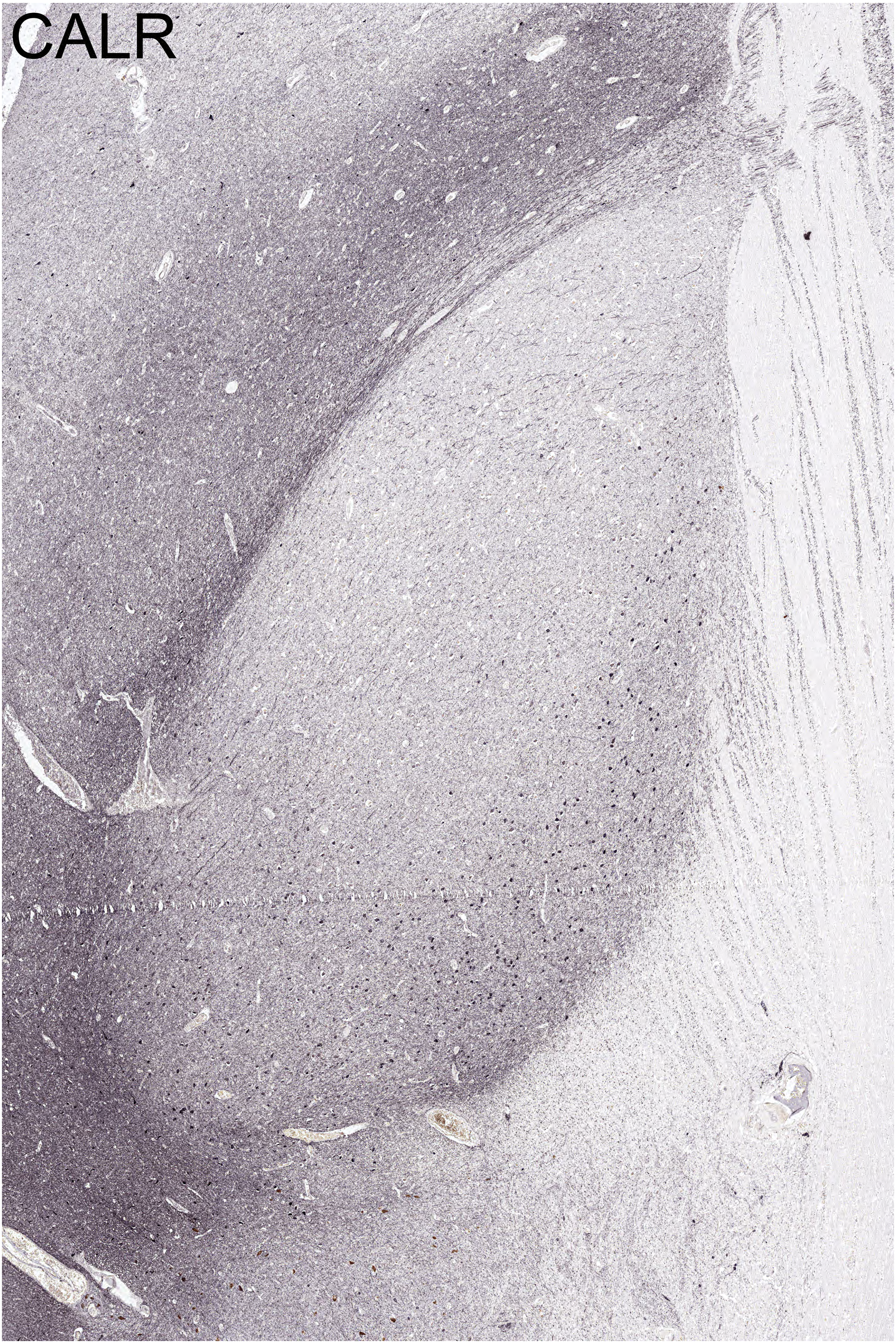
CALR

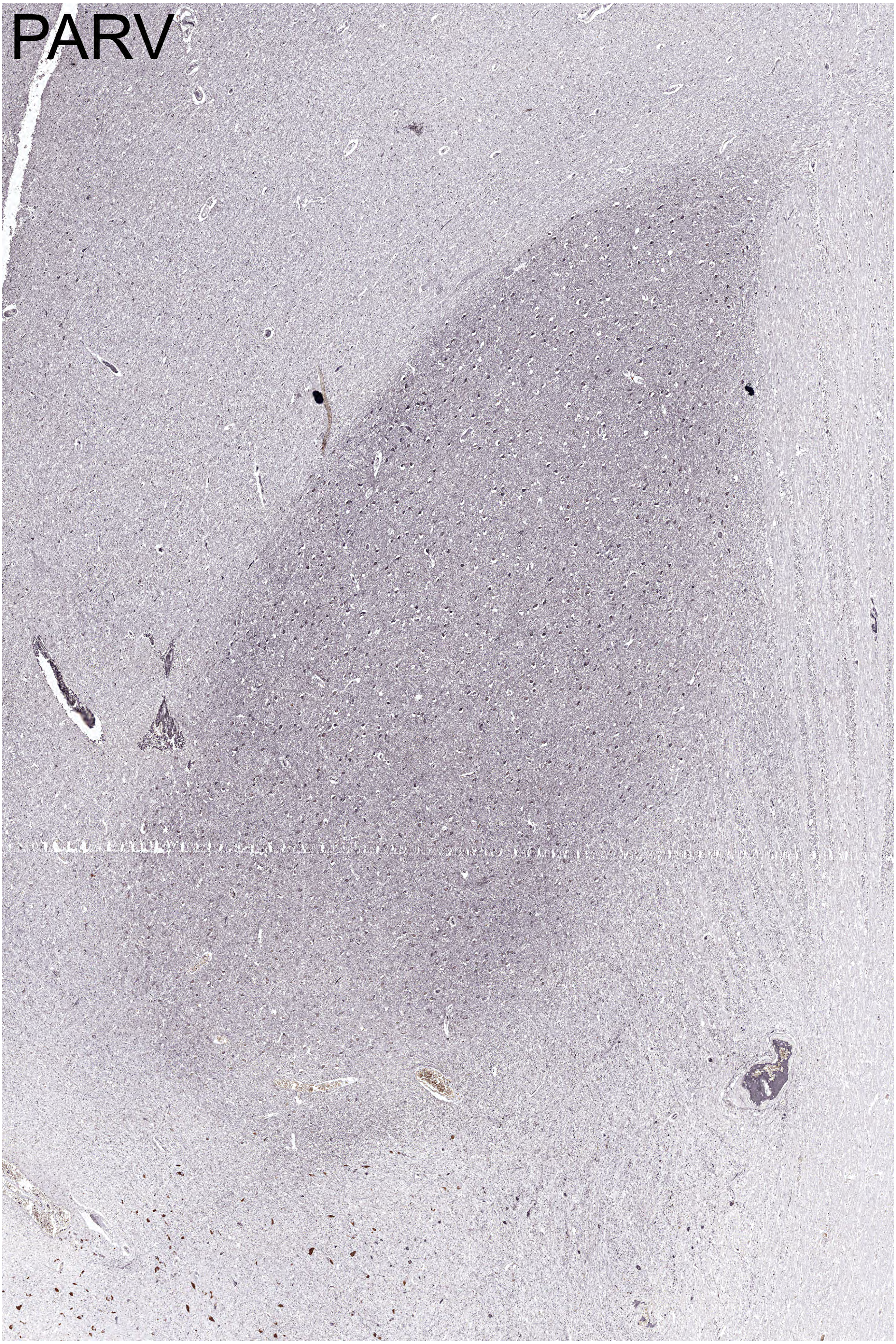
PARV

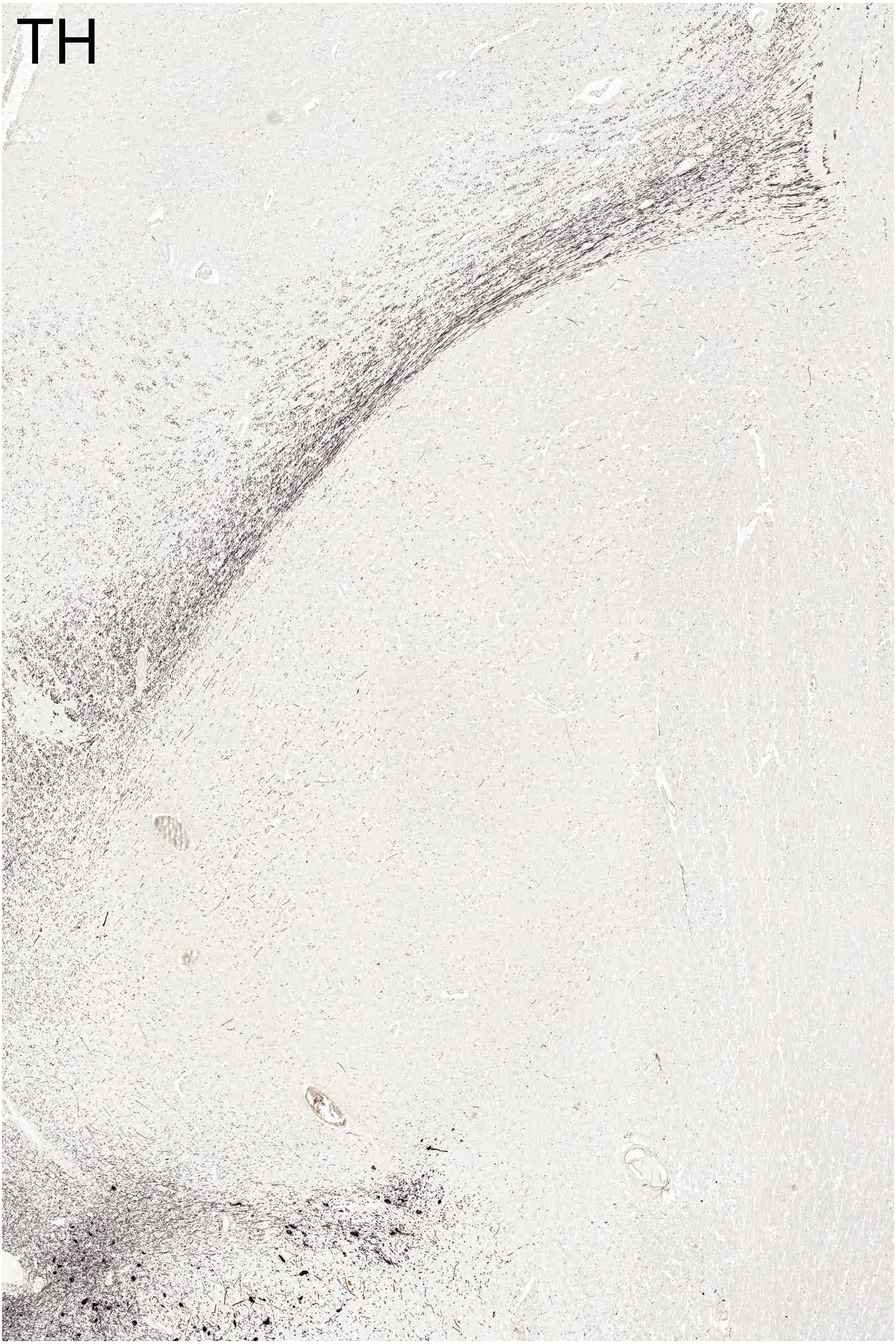
TH

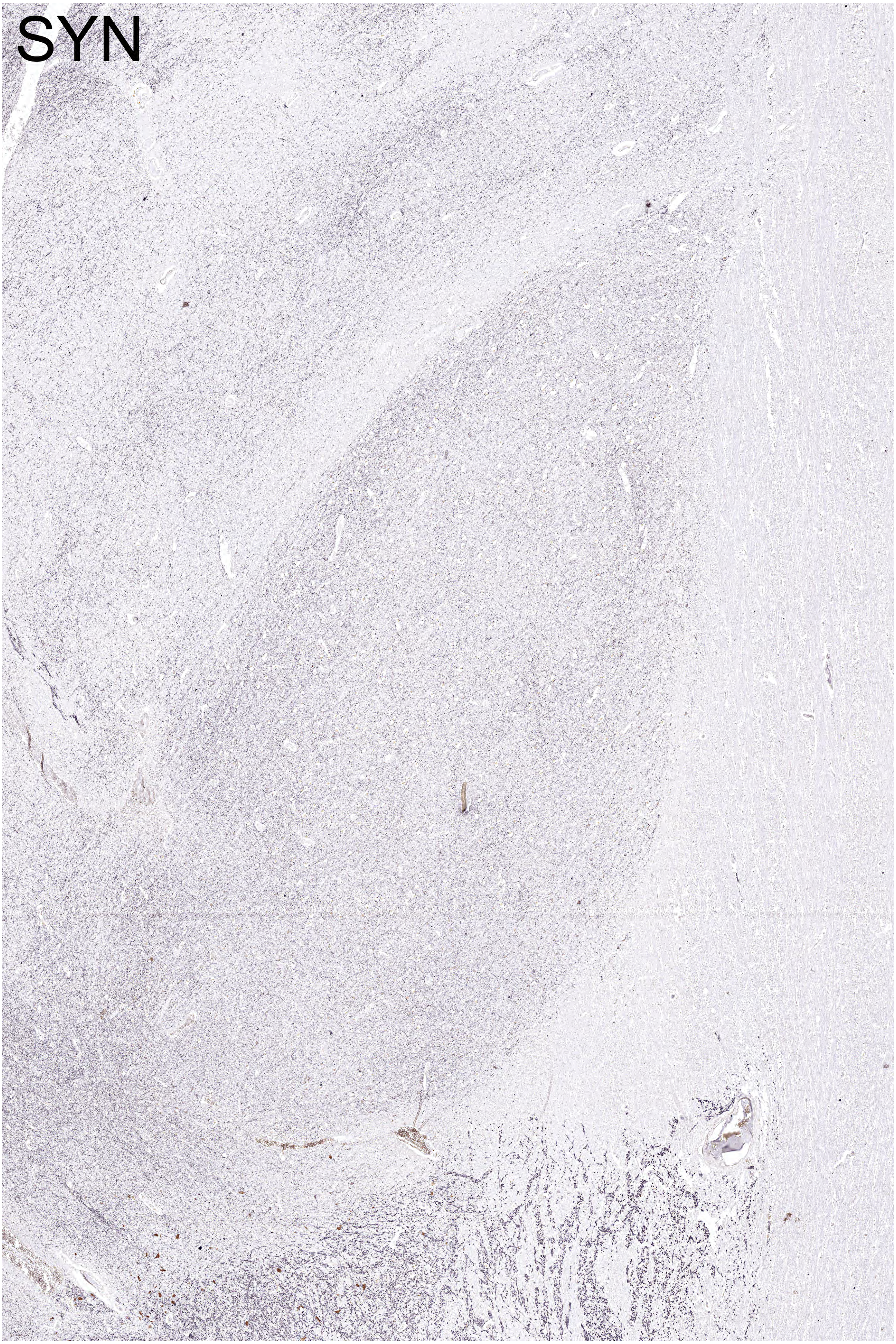
SYN

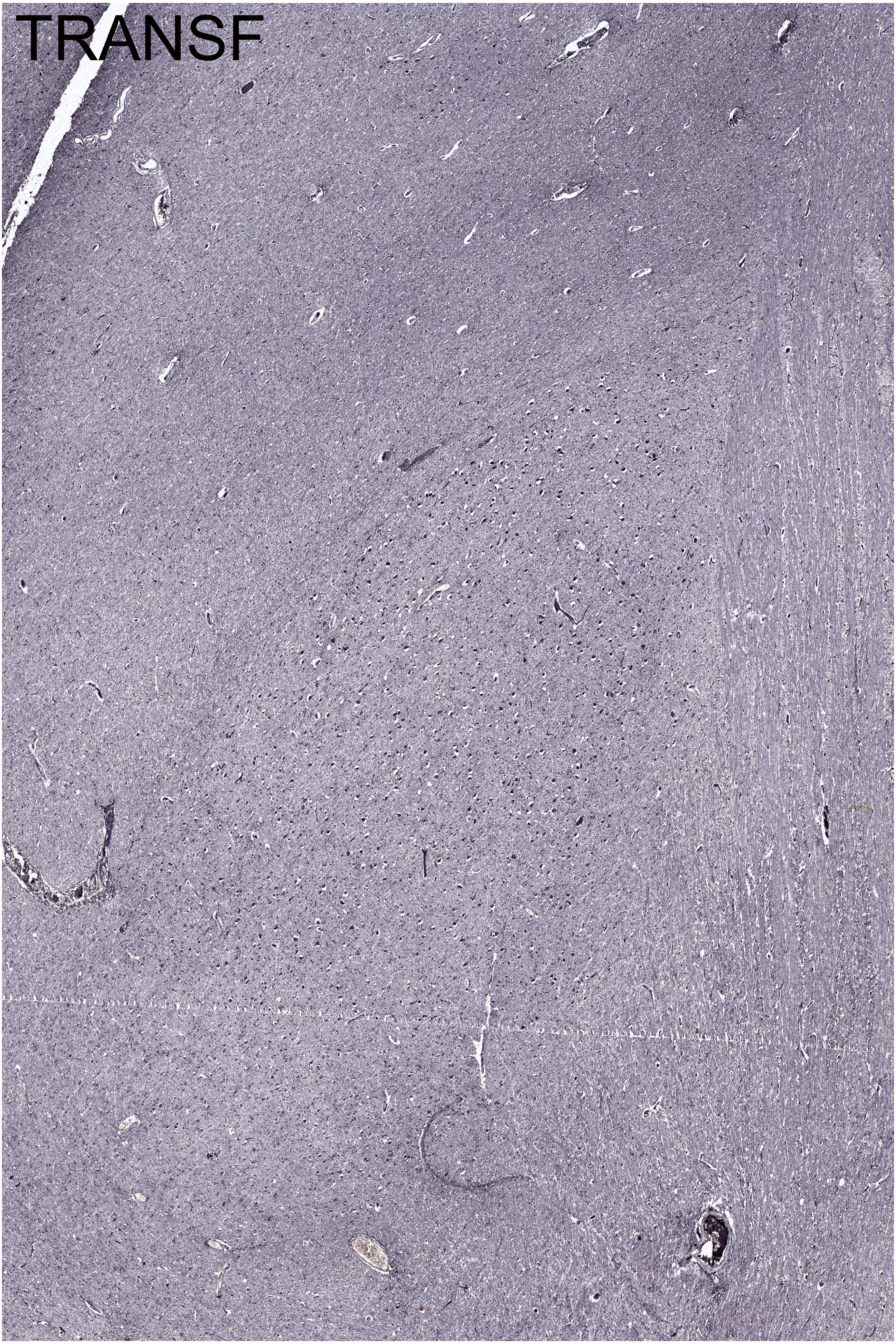
TRANSF

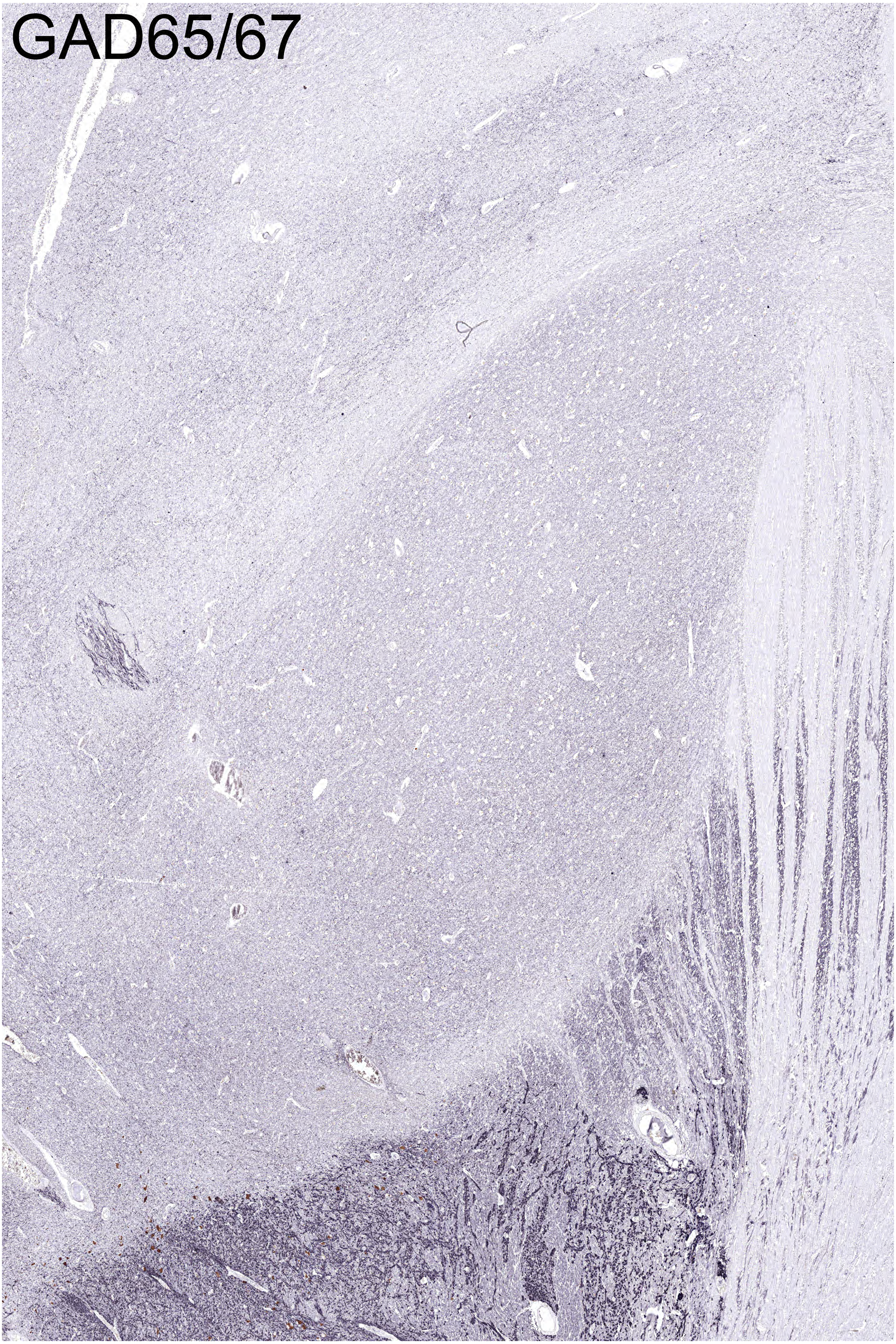
GAD65/67

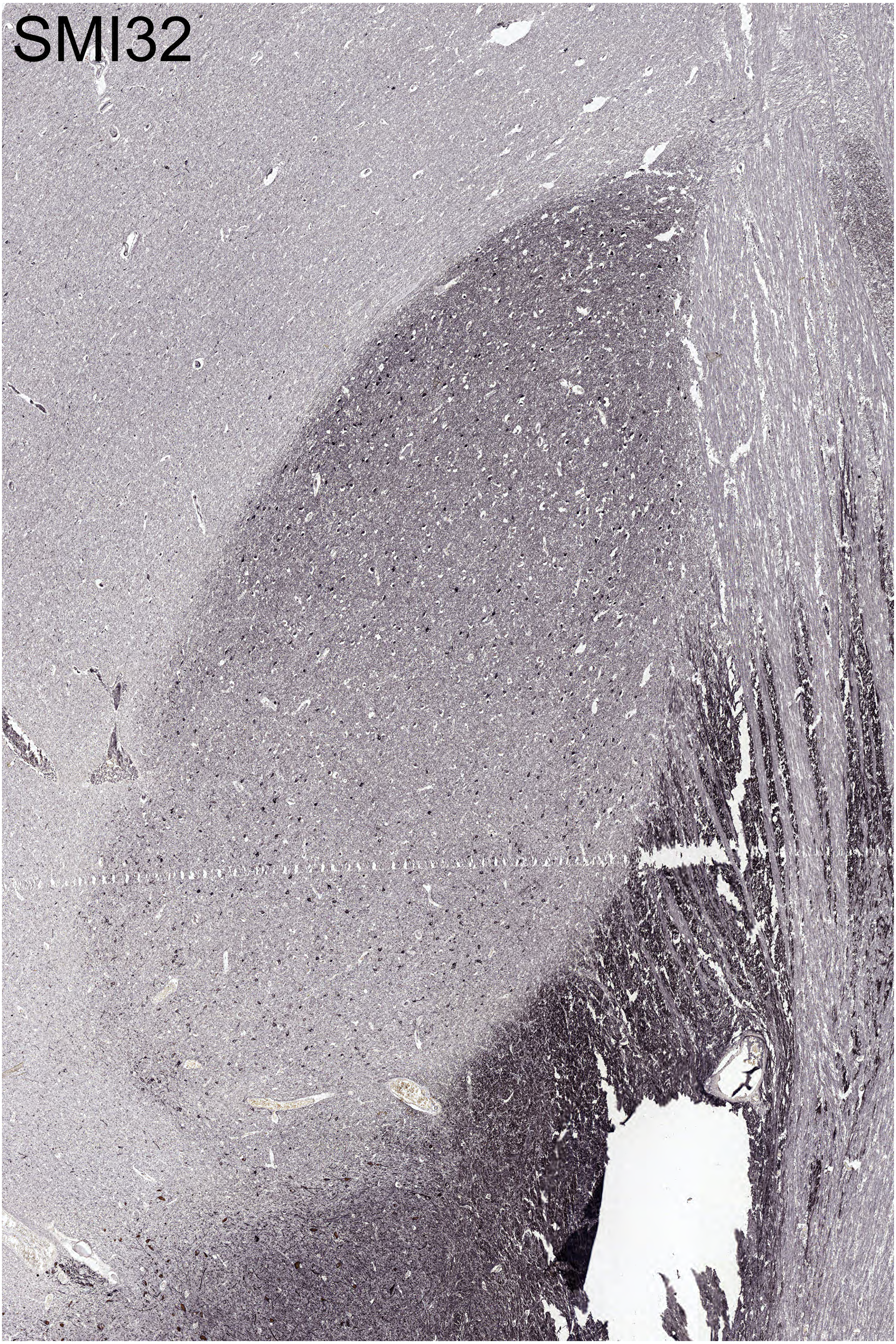
SMI32

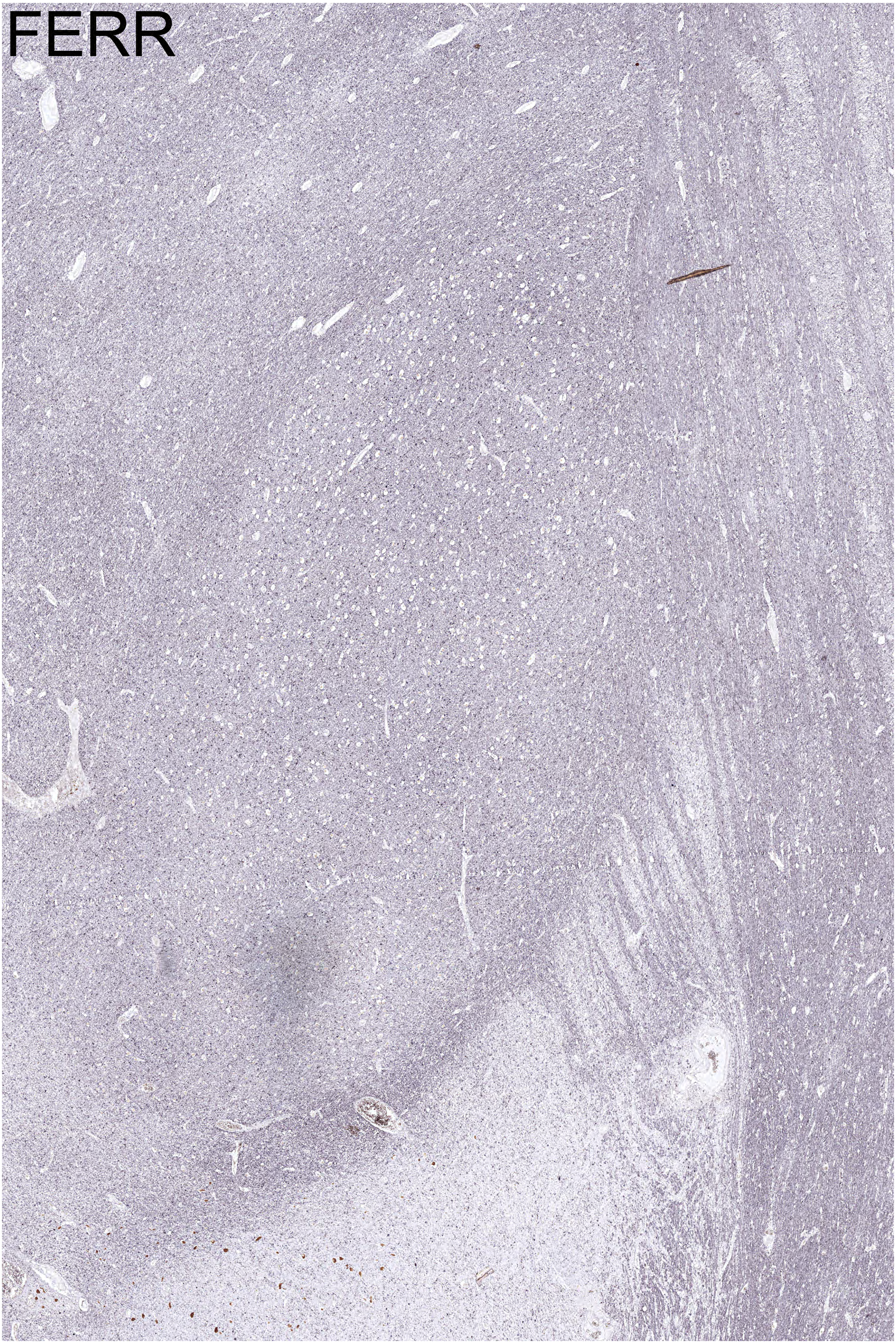
FERR

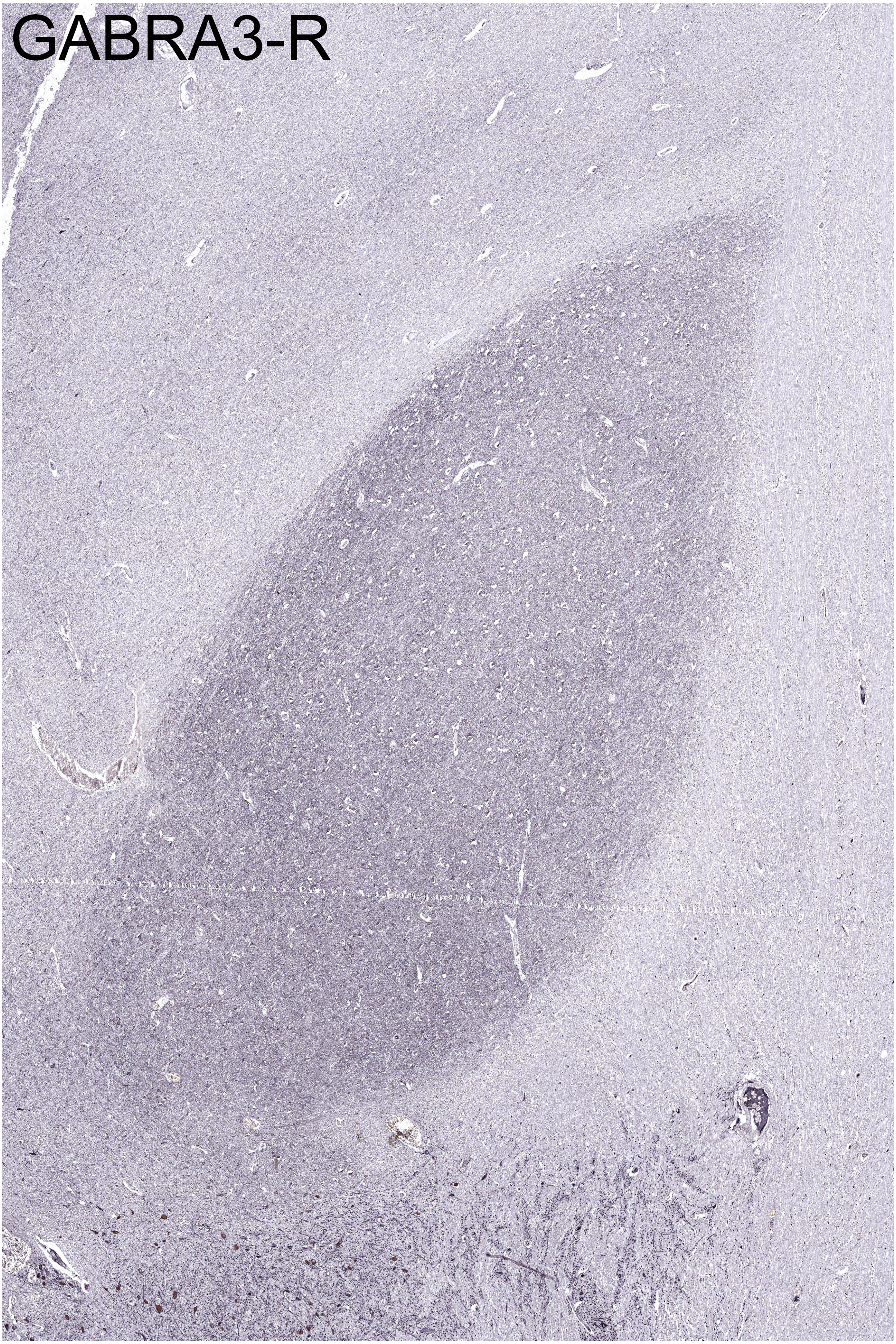
GABRA3-R

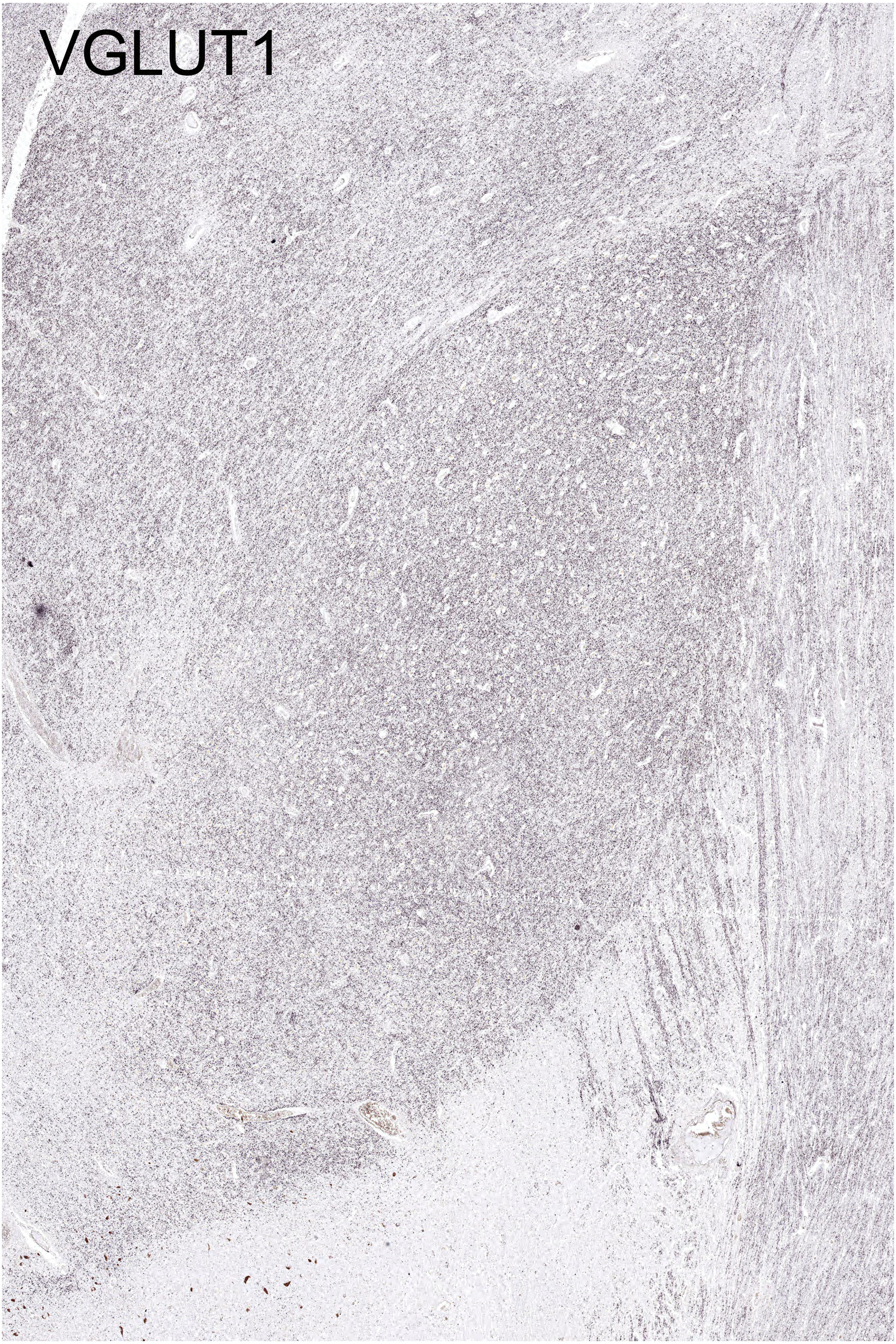
VGLUT1

MBP

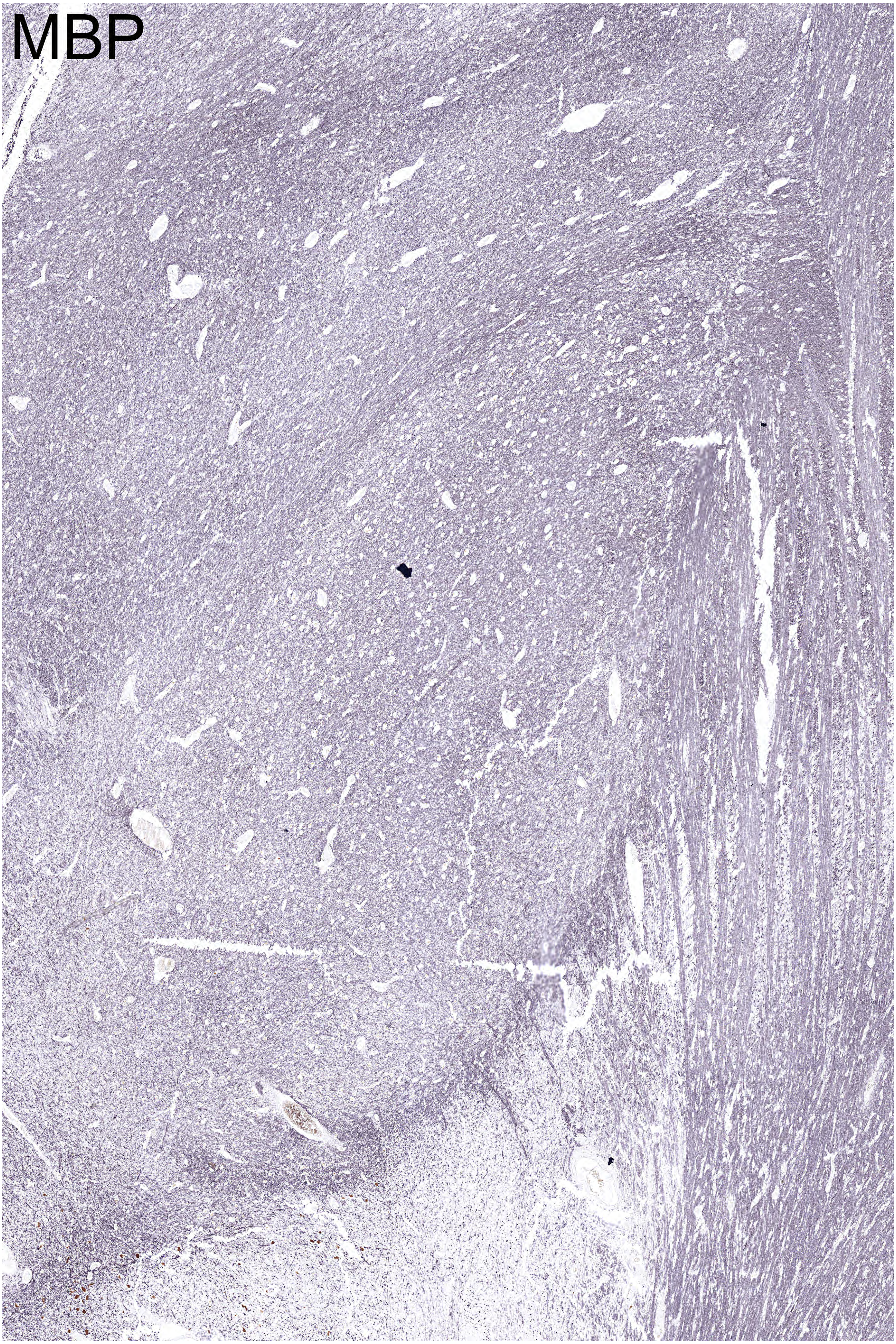

Supplement: Supplementary file 1 — Supplementary material 1 (PDF 20146 kb) [file 429_2019_1960_MOESM1_ESM.pdf]
